# Oleic acid stimulates HC11 mammary epithelial cells proliferation and mammary gland development in peripubertal mice through activation of CD36-Ca^2+^ and PI3K/Akt signaling pathway

**DOI:** 10.18632/oncotarget.24204

**Published:** 2018-01-12

**Authors:** Yingying Meng, Jing Zhang, Cong Yuan, Fenglin Zhang, Qin Fu, Han Su, Xiaotong Zhu, Lina Wang, Ping Gao, Gang Shu, Qingyan Jiang, Songbo Wang

**Affiliations:** ^1^ Guangdong Provincial Key Laboratory of Animal Nutrition Control, College of Animal Science, South China Agricultural University, Guangzhou 510642, P. R. China; ^2^ National Engineering Research Center for Breeding Swine Industry and UBT Lipid Suite Functional Fatty Acids Research Center, South China Agricultural University, Guangzhou 510642, P. R. China

**Keywords:** oleic acid (OA), mammary gland development, CD36-[Ca^2+^]i, PI3K/Akt, peripubertal mice

## Abstract

This study aimed to investigate the effects of oleic acid (OA), a monounsaturated fatty acid, on HC11 mammary epithelial cells proliferation and peripubertal mammary gland development and explore the underlying mechanisms. HC11 cells and C57BL/6J mice were treated with OA. HC11 proliferation, peripubertal mammary gland development, and the involvement of CD36 and PI3K/Akt were assessed. *In vitro*, 100 μM OA significantly promoted HC11 proliferation by increasing Cyclin D1/3 and PCNA expression and decreasing p21 expression. Meanwhile, OA enhanced CD36 expression, elevated [Ca^2+^]_i_ and activated PI3K/Akt signaling pathway. However, knockdown of CD36, chelation of [Ca^2+^]_i_ or inhibition of PI3K eliminated the OA-induced promotion of HC11 proliferation and change in proliferative markers expression. *In vivo*, peripubertal exposure to diet containing 2% OA stimulated mammary duct development, with increased terminal duct end (TDE) and ductal branch. Moreover, dietary OA increased the serum levels of IGF-1 and E2, enhanced the expression of CD36 and Cyclin D1, and activated PI3K/Akt pathway in mammary glands. In conclusion, OA stimulated HC11 cells proliferation and mammary gland development in peripubertal mice, which was associated with activation of CD36-[Ca^2+^]_i_ and PI3K/Akt signaling pathway. These data provided new insights into the stimulation of mammary gland development by dietary oleic acid.

## INTRODUCTION

The mammary glands of mammals are specialized to produce milk for the growth, development and health of mammalian neonates. The development of mammary glands occurs in several different stages, including embryo, puberty, pregnancy, lactation and involution [1]. Ductal elongation and branching of mammary glands occur mainly during puberty, whereas alveolar proliferation and differentiation happen during gestation and lactation [2]. It has been implicated that inhibition of pubertal mammary gland development results in the impairment of development and lactation in the following stages [3, 4]. Thus, ensuring the good development of pubertal mammary glands is critical to their subsequent normal development and function.

Mammary gland development during puberty is influenced by hormones such as estrogen and growth hormone, and the growth factor, insulin-like growth factor-1 (IGF-1) [5]. These factors stimulate the terminal end bud (TEB) or terminal duct end (TDE) formation and ductal elongation and branching by promoting epithelial cell proliferation through the activation of the phosphatidylinositol 3-kinase (PI3K)/ protein kinase B (Akt) signaling pathway [1]. Pubertal mammary gland development is also affected by dietary nutrition [6, 7]. It has been demonstrated that high fat diet intake leads to stunted mammary duct elongation and reduced mammary epithelial cell proliferation in the mammary gland of pubertal C57BL/6 mice [8]. In addition, our recent findings showed that lauric acid, a medium-chain fatty acid (MCFA), stimulated mammary gland development in pubertal mice [9]. Furthermore, long-chain fatty acids (LCFA) such as n-3 and n-6 polyunsaturated fatty acids (PUFA), have been reported to play important roles in the morphological development of the mammary gland during puberty [10]. However, the effects of monounsaturated LCFA on the development of pubertal mammary glands are rarely studied.

Oleic acid (OA), a monounsaturated fatty acid (MUFA) with an 18-carbon atom chain, is the primary fatty acid of olive oil, with approximately 55%-83% of total fatty acids [11]. As the most representative food of a traditional Mediterranean diet, olive oil has beneficial effects on reducing the risk of cardiovascular diseases and metabolic syndrome, preventing several varieties of cancers, and modifying immune and inflammatory responses [12, 13]. It has been suggested that the beneficial effects of olive oil are mainly attributed to its high OA content [14]. Accordingly, it has been implicated that OA have potential role in ameliorating inflammatory and cardiovascular diseases [15–17]. In addition, there is evidence supporting an inverse association between the high consumption of olive oil (rich in OA) and the risk of breast cancer [18]. Furthermore, OA has been demonstrated to induce the proliferation of human vascular smooth muscle cells [19, 20] and rat islet β cells [21]. It has been shown that the fatty acid translocase (FAT/CD36), a receptor and transport protein of LCFA [22, 23], is involved in OA-mediated various functions such as taste transduction [24], lipid sensing in the gut and brain [25], ovarian angiogenesis and folliculogenesis [26], and cell proliferation [19].

However, the role of OA in mammary epithelial cells proliferation and pubertal mammary gland development has not been investigated yet. Thus, this study was designed to investigate the effects of OA on HC11 proliferation and mammary gland development. In addition, we sought to explore the underlying mechanism in this process, including the contribution of CD36, intracellular calcium ([Ca^2+^]_i_), and the related intracellular signaling pathway. Our data showed that OA stimulated HC11 proliferation and mammary gland development through the activation of CD36-[Ca^2+^]_i_ and PI3K/Akt signaling pathway.

## RESULTS

### OA enhanced the proliferation of HC11 and the expression of CD36

**Figure 1:**
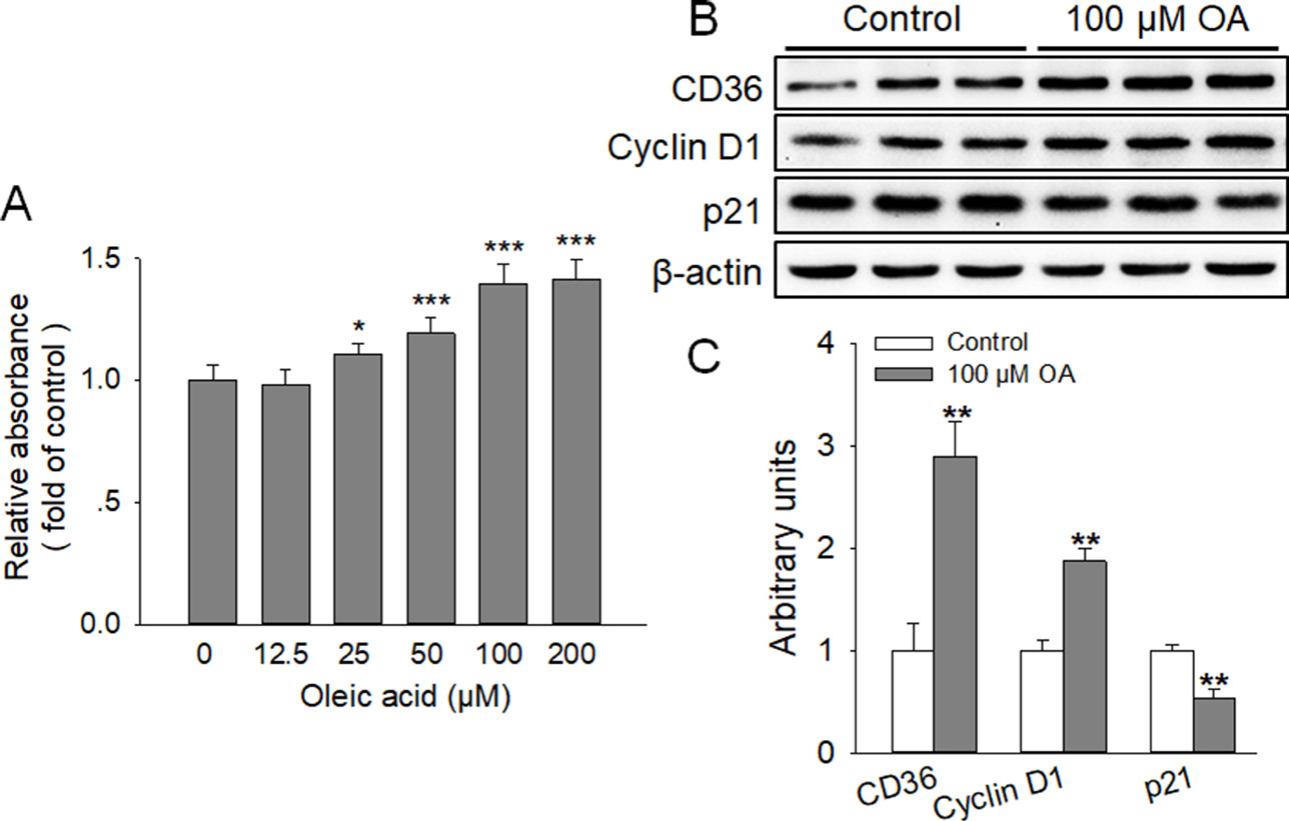
OA enhanced the proliferation of HC11 and the expression of CD36 (**A**) Effect of various concentrations of OA (0, 12.5, 25, 50, 100, and 200 μM) on the proliferation of HC11 after a 4-day culture was determined by MTT analysis. (**B**) Western blot analysis of proliferative markers (Cyclin D1 and p21) and CD36 in HC11 after a 4-day culture. β-actin was used as the loading control. (**C**) Mean ± SEM of immunoblotting bands of CD36, Cyclin D1 and p21, and the intensities of the bands were expressed as the arbitrary units. ^*^P < 0.05, ^**^P < 0.01 and ^***^P < 0.001 versus the 0 μM OA (Control) group.

**Figure 2:**
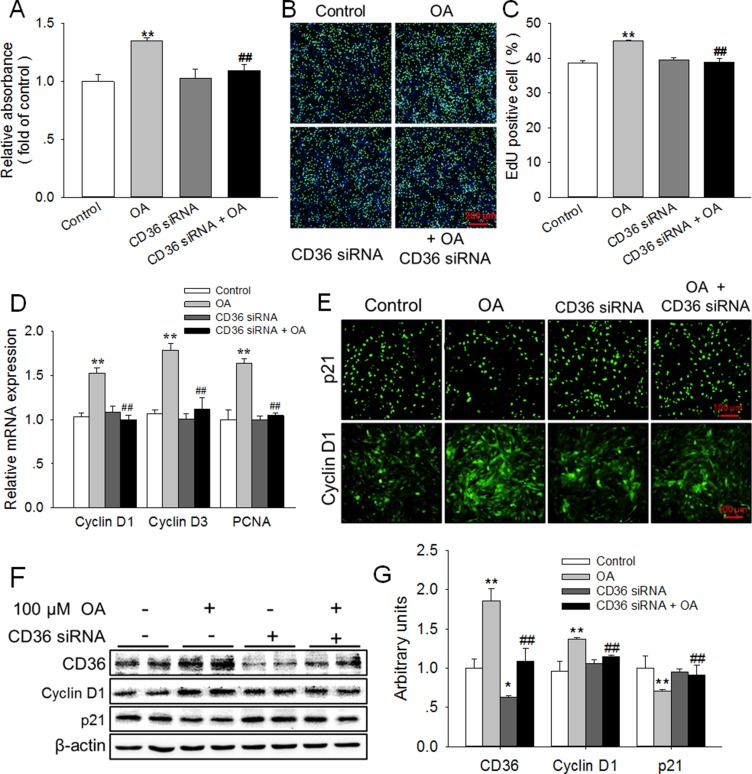
Knockdown of CD36 eliminated the enhancement of HC11 proliferation induced by OA (**A**, **B**) Effects of 100 μM OA and/or CD36 siRNA on HC11 proliferation was determined using MTT analysis (**A**) and EdU incorporation assay (**B**). The nuclei were stained with Hoechst and the scale bar = 200 μm. (**C**) Analysis of EdU positive cell percentage in panel B. (**D**) The relative mRNA expression level of Cyclin D1, Cyclin D3, and PCNA in response to 100 μM OA and/or CD36 siRNA. (**E**) The representative immunofluorescence staining of Cyclin D1 and p21 in the presence of 100 μM OA and/or CD36 siRNA. Scale bar = 100 μm. (**F**) Western blot analysis of CD36, Cyclin D1 and p21 in HC11 after a 4-day culture. β-actin was used as the loading control. (**G**) Mean ± SEM of immunoblotting bands of CD36, Cyclin D1, and p21. The intensities of the bands were expressed as the arbitrary units. ^**^P < 0.01 versus the Control group, ^##^*P* < 0.01 versus the 100 μM OA group.

To assess the effect of OA on the proliferation of HC11, cells were incubated in RPMI-1640 supplemented with various concentrations (0, 12.5, 25, 50, 100, and 200 μM) of OA for 4 days. The results of MTT assay demonstrated that OA significantly stimulated HC11 proliferation in a dose-dependent manner, with the similar promotive effects observed at 100 and 200 μM OA (Figure 1A). Thus, 100 μM OA was selected and used in the following studies. Meanwhile, EdU incorporation assay was conducted to examine the effects of 100 μM OA on the percentage of cells undergoing DNA replication. The findings revealed that the percentage of EdU positive cell was significantly (*P* < 0.01) elevated by OA (Figure 2B, 2C). In addition, immunofluorescence staining of proliferative markers, such as Cyclin D1 and p21, revealed that OA remarkably enhanced the expression of Cyclin D1 and attenuated the expression of p21 (Figure 2E). In agreement, the protein expression levels of Cyclin D1 and p21 were markedly (*P* < 0.01) increased and decreased by OA, respectively (Figure 1B, 1C). These data showed that OA stimulated HC11 proliferation by modulating (increasing or decreasing) the expression of the proliferative markers. Furthermore, the protein expression of CD36 was profoundly (*P* < 0.01) increased by 100 μM OA (Figure 1B, 1C), implying the possible participation of CD36 in this process. We also found that 100 μM OA had no effect on GPR120 expression (data not shown).

### Knockdown of CD36 eliminated the enhancement of HC11 proliferation induced by OA

To further determine whether CD36 was involved in OA-enhanced HC11 proliferation, CD36 siRNA was used to knockdown the expression of CD36 in this study. Indeed, as shown in Figure 2F, 2G, the protein expression of CD36 was significantly (*P* < 0.05) decreased by CD36 siRNA. The knockdown of CD36 with siRNA alone had no effect on HC11 proliferation. However, CD36 siRNA eliminated the enhancement of HC11 proliferation induced by 100 μM OA (Figure 2A). Meanwhile, the increase of the percentage of cells undergoing DNA replication induced by OA was reversed by CD36 siRNA (Figure 2B, 2C). In addition, the OA-induced elevation of the mRNA expression of proliferative markers, including Cyclin D1, Cyclin D3 and proliferating cell nuclear antigen (PCNA), was abolished by CD36 siRNA (Figure 2D). Furthermore, the results of immunofluorescence staining (Figure 2E) demonstrated that the increase in Cyclin D1 expression and decrease in p21 expression induced by 100 μM OA were also eliminated by CD36 siRNA. Similarly, the elevated protein expression of Cyclin D1 and the reduced protein level of p21 induced by 100 μM OA were reversed by CD36 siRNA (Figure 2F, 2G). These results demonstrated that CD36 knockdown eliminated the promotive effects of OA on HC11 proliferation, thereby indicating the essential role of CD36 in this process.

### OA increased [Ca^2+^]^i^ and activated the PI3K/Akt signaling pathway in a CD36-dependent manner

**Figure 3:**
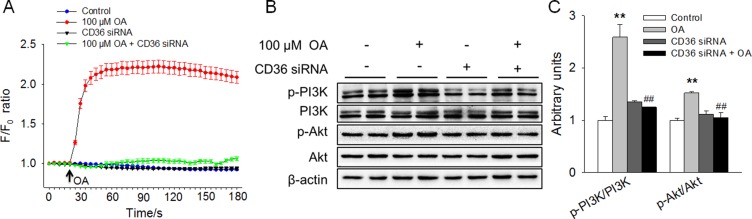
OA increased [Ca^2+^]_i_ and activated the PI3K/Akt signaling pathway in a CD36-dependent manner (**A**) The changes in [Ca^2+^]_i_ in response to OA and/or CD36 siRNA in HC11 cells. (**B**) Western blot analysis of p-PI3K, PI3K, p-Akt, and Akt in HC11 after a 4-day culture in the presence of OA and/or CD36 siRNA. β-actin was used as the loading control. (**C**) Mean ± SEM of immunoblotting bands of p-PI3K/PI3K and p-Akt/Akt, and the intensities of the bands were expressed as the arbitrary units. ^**^P < 0.01 versus the Control group, ^##^*P* < 0.01 versus the 100 μM OA group.

The possible intracellular signals involved in OA-enhanced HC11 proliferation were further explored. First, we assessed whether Ca^2+^ signaling was associated with OA-stimulated HC11 proliferation by determining the level of [Ca^2+^]_i_. The results revealed that OA remarkably elevated the [Ca^2+^]_i_ level. However, the elevation of [Ca^2+^]_i_ level induced by 100 μM OA was abolished by CD36 siRNA, which alone had no effect on [Ca^2+^]_i_ (Figure 3A). Besides the increase in the [Ca^2+^]_i_ concentration, we further examined the possible involvement of the intracellular PI3K/Akt signaling pathway in OA-stimulated HC11 proliferation. We found that 100 μM OA significantly (*P* < 0.01) increased the ratios of the p-PI3K/PI3K and p-Akt/Akt, indicating the activation of the PI3K/Akt signaling pathway (Figure 3B, 3C). Interestingly, the activation of the PI3K/Akt signaling pathway induced by OA was eliminated by CD36 siRNA (Figure 3B, 3C). These results demonstrated that OA increased [Ca^2+^]_i_ and activated the PI3K/Akt signaling pathway in a CD36-dependent manner and suggested that the activation of CD36-[Ca^2+^]_i_ and the intracellular PI3K/Akt signaling pathway might be involved in OA-promoted HC11 proliferation.

### Chelation of [Ca^2+^]^i^ reversed OA-induced activation of PI3K/Akt and promotion of HC11 proliferation

**Figure 4:**
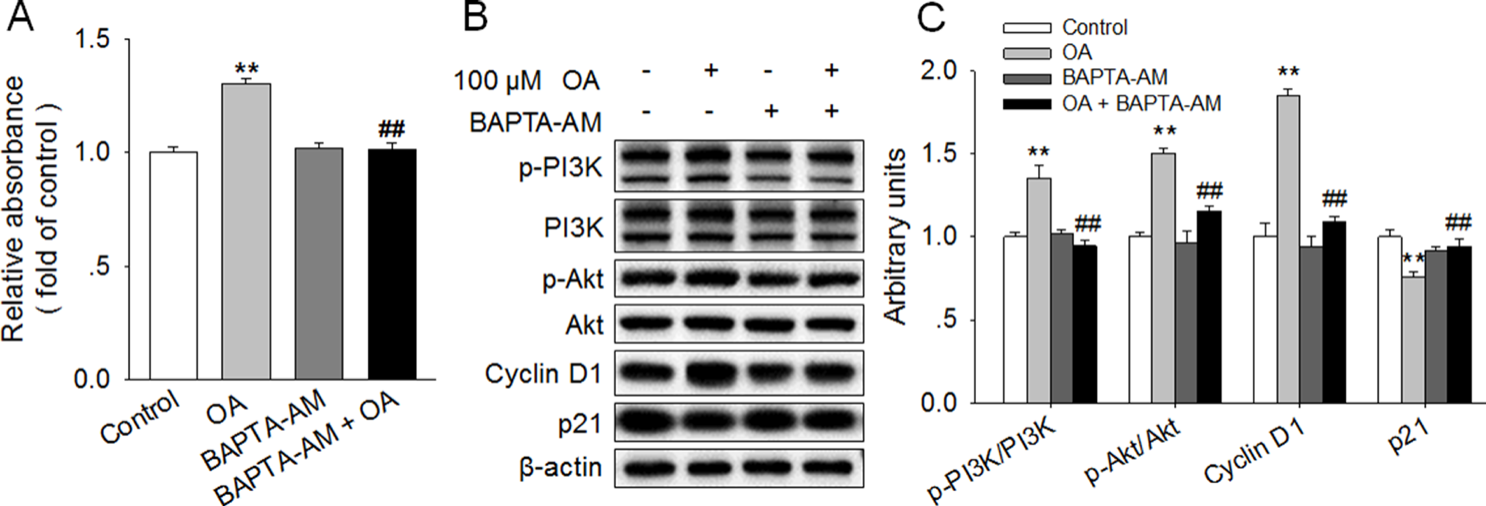
Chelation of [Ca^2+^]_i_ reversed the OA-induced activation of PI3K/Akt and promotion of HC11 proliferation (**A**) Effects of 100 μM OA and/or 2 μM BAPTA-AM on HC11 proliferation by using MTT analysis. (**B**) Western blot analysis of p-PI3K, PI3K, p-Akt, Akt, Cyclin D1, and p21 in HC11 after a 4-day culture in the presence of 100 μM OA and/or 2 μM BAPTA-AM. β-actin was used as the loading control. (**C**) Mean ± SEM of immunoblotting bands of p-PI3K/PI3K, p-Akt/Akt, Cyclin D1 and p21. The intensities of the bands were expressed as the arbitrary units. ^**^P < 0.01 versus the Control group, ^##^*P* < 0.01 versus the 100 μM OA group.

To further elucidate the role of [Ca^2+^]_i_ signaling in OA-stimulated cell proliferation of HC11, BAPTA-AM, a selective and membrane-permeable calcium chelator, was applied in this study. BAPTA-AM alone had no effect on HC11 proliferation. However, BAPTA-AM abolished the enhancement of HC11 proliferation induced by 100 μM OA (Figure 4A). Accordingly, the increased protein expression of Cyclin D1 and the decreased protein level of p21 induced by 100 μM OA were also eliminated by BAPTA-AM. In addition, we found that the significantly increased ratios of p-PI3K/PI3K and p-Akt/Akt in response to 100 μM OA were reversed by 2 μM BAPTA-AM (Figure 4B, 4C), suggesting a link between the increase in [Ca^2+^]_i_ level and the activation of the PI3K/Akt signaling pathway. These results strongly suggested that the increase in [Ca^2+^]_i_ and subsequent activation of PI3K/Akt signaling pathway were responsible for the OA-promoted HC11 proliferation.

### Inhibition of PI3K/Akt totally blocked the promotion of HC11 proliferation induced by OA

**Figure 5:**
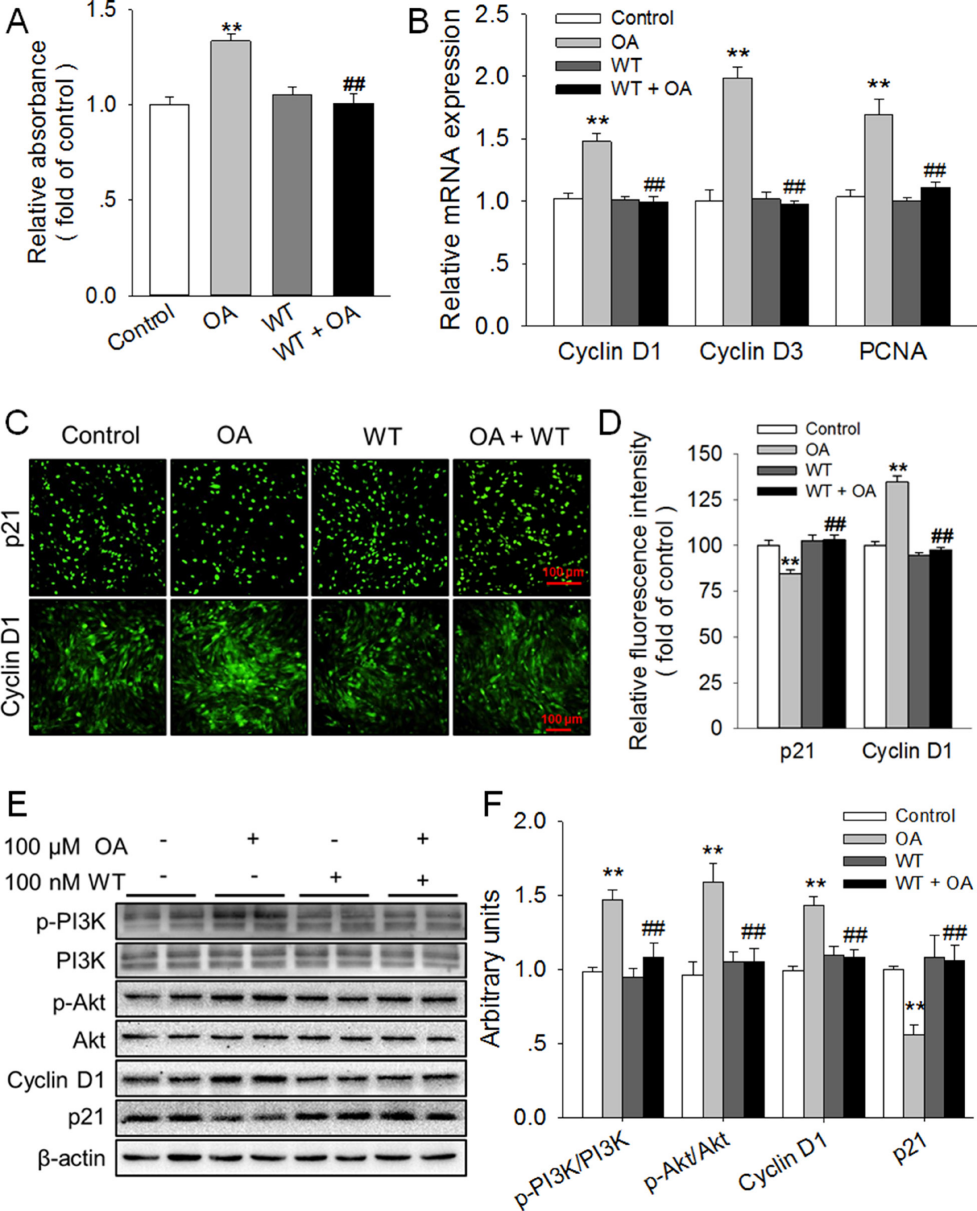
Inhibition of PI3K/Akt totally blocked the promotion of HC11 proliferation induced by OA (**A**) Effect of Wortmannin (WT), an inhibitor of PI3K, on the proliferation of HC11 after a 4-day incubation was determined by MTT assay. (**B**) The relative mRNA expression level of Cyclin D1, Cyclin D3, and PCNA in response to 100 μM OA and/or 100 nM WT. (**C**) Representative immunofluorescence staining of Cyclin D1 and p21 in the presence of 100 μM OA and/or 100 nM WT. Scale bar = 100 μm. (**D**) Analysis of the relative fluorescence intensity in panel (**C). (E**) Western blot analysis of p-PI3K, PI3K, p-Akt, Akt, Cyclin D1 and p21 in HC11 after a 4-day culture in the presence of 100 μM OA and/or 100 nM WT. β-actin was used as the loading control. (**F**) Mean ± SEM of immunoblotting bands of p-PI3K/PI3K, p-Akt/Akt, Cyclin D1, and p21, and the intensities of the bands were expressed as the arbitrary units. ^**^P < 0.01 versus the Control group, ^##^*P* < 0.01 versus the 100 μM OA group.

To further verify the role of the PI3K/Akt signaling pathway in OA-promoted HC11 proliferation, Wortmannin (WT), a potent and selective inhibitor of PI3K, was applied to inhibit the activation of PI3K and thus prevent the activation of Akt in this study. As expected, we found that the significant (*P* < 0.01) increase of p-PI3K/PI3K and p-Akt/Akt ratios in response to 100 μM OA was reversed by 100 nM WT (Figure 5E, 5F). Meanwhile, the OA-induced promotion of HC11 proliferation was totally blocked by WT, which alone had no influence on the proliferation of HC11 (Figure 5A). In good agreement, the significant elevation of the mRNA level of Cyclin D1, Cyclin D3 and PCNA induced by 100 μM OA was eliminated by WT (Figure 5B). In addition, by using immunofluorescence staining, we found that the remarkably (*P* < 0.01) enhanced expression of Cyclin D1 (Figure 5C, 5D) and the significantly (*P* < 0.01) reduced expression of p21 induced by 100 μM OA were also abolished by WT (Figure 5B, 5C). Furthermore, the OA-induced increase in Cyclin D1 protein expression and decrease in p21 protein expression was reversed by WT (Figure 5E, 5F). These results strongly suggested that the activation of the PI3K/Akt signaling pathway participated in the OA-promoted HC11 proliferation.

### Peripubertal exposure to diet containing 2% OA promoted mammary duct growth of mice

**Figure 6:**
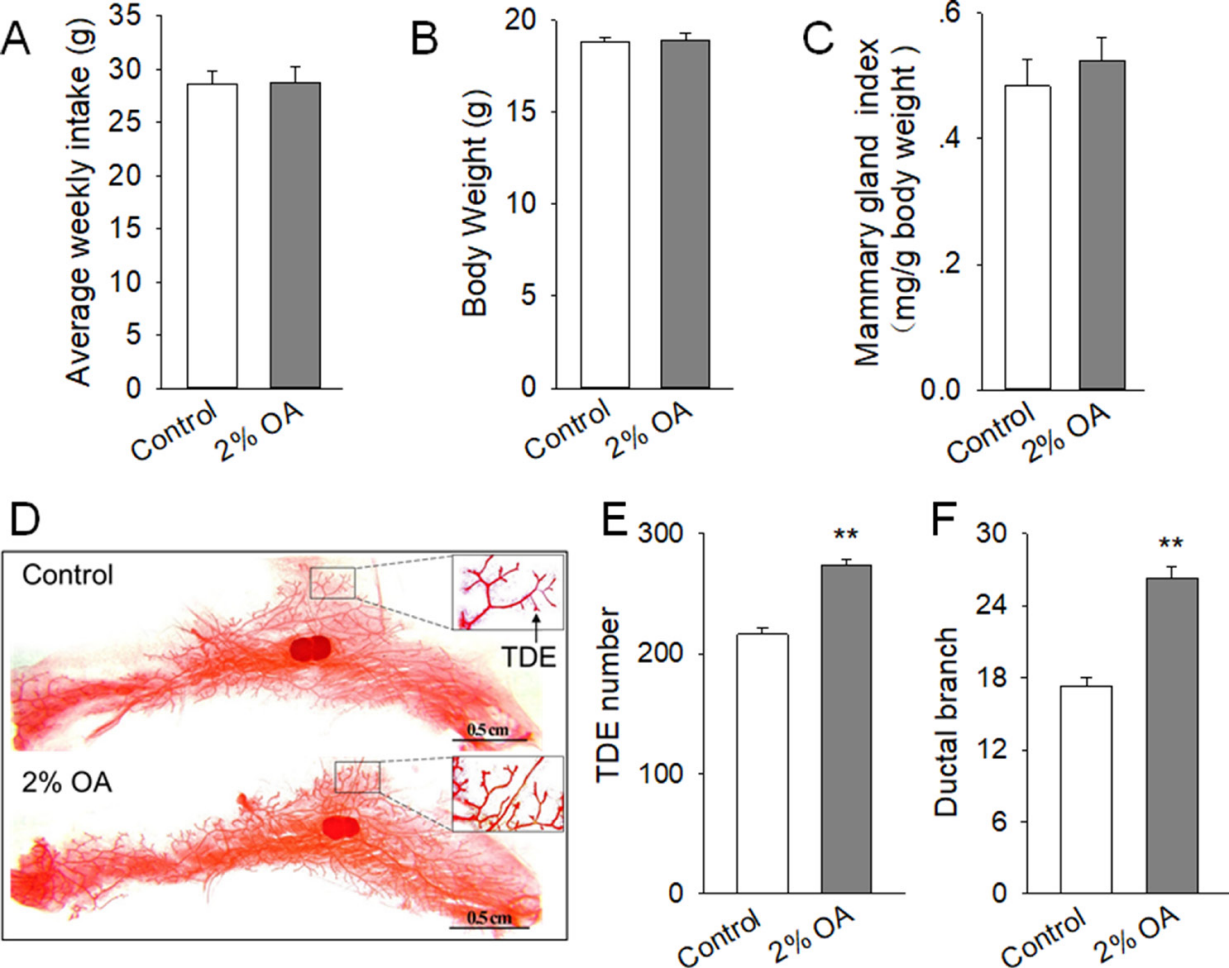
Effects of peripubertal exposure to diet containing 2% OA on mammary duct growth of mice (**A–C**) Effects of dietary 2% OA on average weekly food intake (A), body weight (B) and mammary gland index (C). (**D**) Representative images of whole mount staining of the left side of mammary gland of the control and 2% OA treated mice. Arrows indicate the TDE and ductal branch. The scale bars are shown in the images. (**E**–**F)**. Effects of peripubertal exposure to diet containing 2% OA on TDE number (E) and ductal branch (F) in the mammary gland. ^**^P < 0.01 versus the Control group.

To determine whether OA could promote mammary gland development *in vivo*, 4-week-old mice were treated with control diet or control diet containing 2% OA for 5 weeks. The average weekly food intake (Figure 6A) and body weight (Figure 6B) were comparable between the control and 2% OA-treated mice. Meanwhile, the mammary gland index (mg/g body weight) of the fourth pair was not significantly altered between the 2% OA-treated and control mice (Figure 6C). However, noticeable morphological changes in mammary glands were observed in OA-treated mice. Whole mount staining revealed that the mammary gland of 2% OA-treated mice contained increased TDE number and ductal branch, with a similar size to that of the control mice (Figure 6D–6F). These results suggested that peripubertal exposure to diet containing 2% OA stimulated the mammary duct growth of mice.

Peripubertal exposure to diet containing 2% OA increased serum level of IGF-1 and E2, enhanced expression of CD36 and Cyclin D1, and activated the PI3K/Akt pathway in mice mammary gland

**Figure 7:**
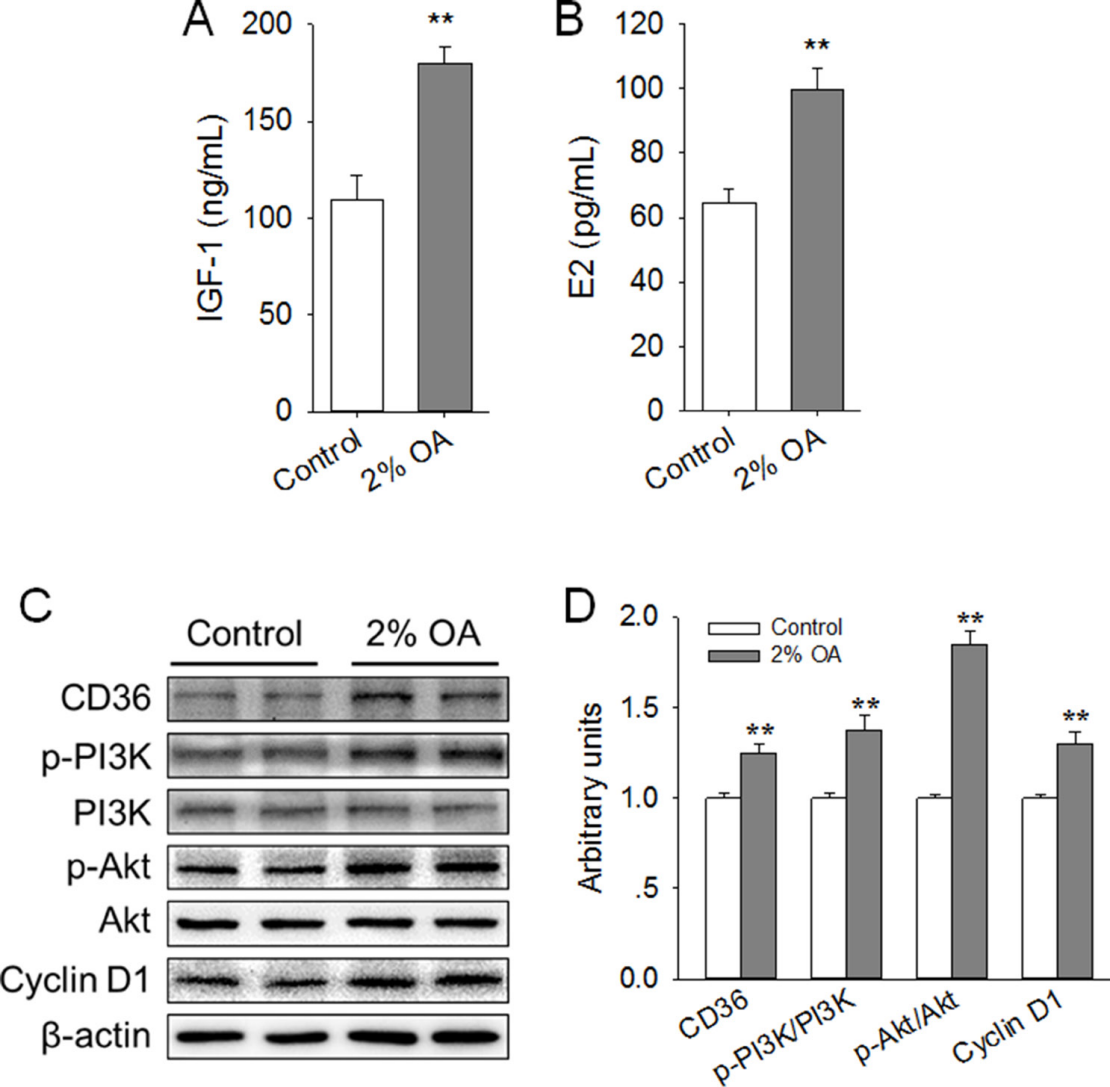
Effects of peripubertal exposure to diet containing 2% OA on the serum level of IGF-1 and E2, expression of CD36 and Cyclin D1, and activation of PI3K/Akt in the right side of the mammary gland of mice (**A**–**B**) Effects of dietary 2% OA on the serum levels of IGF-1 (A) and E2 (B) (*n* = 10). (**C**) Western blot analysis of CD36, p-PI3K, PI3K, p-Akt, Akt and cyclin D1 in the mammary gland of pubertal mice (*n* = 6). β-actin was used as the loading control. (**D**) Mean ± SEM of immunoblotting bands of CD36, p-PI3K/PI3K, p-Akt/Akt and Cyclin D1, and the intensities of the bands were expressed as the arbitrary units. ^**^P < 0.01 versus the Control group.

We further explored the possible mechanism by which dietary OA enhanced mice mammary gland development. The results showed that the serum levels of IGF-1 (Figure 7A) and E2 (Figure 7B) were significantly (*P* < 0.01) increased by dietary 2% OA. Meanwhile, in line with the *in vitro* findings, we found that peripubertal exposure to diet supplemented with 2% OA significantly (*P* < 0.01) promoted the expression of CD36 (Figure 7C, 7D). In addition, dietary 2% OA significantly (*P* < 0.01) elevated the ratios of p-PI3K/ PI3K and p-Akt/Akt, indicating the activation of the PI3K/Akt signaling pathway (Figure 7C, 7D). Moreover, the protein expression of proliferative marker Cyclin D1 was also markedly (*P* < 0.01) increased by dietary 2% OA (Figure 7C, 7D). These results suggested that the enhancement of CD36 expression, activation of the PI3K/Akt signaling pathway and subsequent increase in Cyclin D1 expression might be associated with the stimulated mammary gland development induced by peripubertal exposure to diet containing 2% OA.

## DISCUSSION

In this study, we determined that OA, a MUFA, stimulated HC11 cells proliferation and peripubertal mammary gland development of mice through the activation of CD36-Ca^2+^ and PI3K/Akt signaling pathway. OA has been reported to stimulate the cell proliferation of human vascular smooth muscle cells [19, 20] and rat islet β cells [21]. In line with previous findings, our results demonstrated that OA promoted HC11 proliferation, with increased percentage of cells undergoing DNA replication. Meanwhile, we detected the expression of proliferative markers such as Cyclin D1/3, p21, and PCNA, and found that OA increased the expression of Cyclin D1/3 and PCNA, as well as decreased the expression of p21, the inhibitor of cyclin-dependent kinase. In agreement, dietary OA stimulated mammary duct growth by increasing the TDE number and ductal branch. In addition, the protein expression of Cyclin D1 in the mammary gland was also elevated by peripubertal exposure to diet containing 2% OA. Furthermore, the serum levels of IGF-1 and E2, which are involved in promoting pubertal mammary gland development [5], were significantly elevated by dietary OA. Thus, these findings showed that OA stimulated HC11 proliferation and mammary duct growth by regulating the serum level of proliferative hormones (IGF-1 and E2) and the expression of the proliferative markers. The effects of OA on mammary gland development might be due to both direct and indirect manner. And the further investigations should be conducted in subsequent studies to elucidate how OA increases the serum level of E2 and IGF-1. In contrast, OA has also been reported to have no significant effect on bovine granulosa cells proliferation, the proportion of cells at S phase, and the mRNA expression of proliferative marker PCNA [27]. Similarly, exogenous supplementation with low concentrations of OA (10 μM) had no notable effects on breast cancer cell proliferation [28]. Moreover, numerous studies have demonstrated an inhibition in cell proliferation induced by OA in different tumor cell lines [29]. The completely different effects of OA on cell proliferation might be attributed to the various cell types and culture conditions.

Similar to the effects of OA, we previously showed that lauric acid, a MCFA, stimulated HC11 proliferation and pubertal mammary gland development [9]. On the contrary, our recently published data demonstrated that stearic acid, a saturated LCFA with 18 carbons, inhibited HC11 proliferation and pubertal mammary gland development [30]. In addition, dietary *trans*-10, *cis*-12 conjugated linoleic acid (CLA), but not cis-9, trans-11 CLA, stimulates mammary gland growth in ovariectomized pubertal mice [31]. Furthermore, the morphological development of mammary glands during puberty is influenced by lifelong exposure to n-3 PUFA [10]. These results suggested that various fatty acids might exhibit distinct effects on mammary epithelial cell proliferation and pubertal mammary gland development, depending on the different fatty acid structures, including carbon chain length, double bond number, double bond position and the isomer.

We further explored the possible mechanisms by which OA induced promotive effects on HC11 proliferation and peripubertal mammary gland development. It has been well documented that CD36 plays a pivotal role in the sensing, transfer, and metabolism of LCFA [22]. To clarify the role of CD36 in OA-stimulated HC11 proliferation and peripubertal mammary gland development, we first examined the expression of CD36 in response to OA treatment. The results demonstrated that CD36 expression was increased by OA treatment in both HC11 cells and mammary gland. In line with our results, it was reported that OA or olive oil treatment increased CD36 expression in bovine granulosa cells [27] and mice liver [32]. In contrast, CD36 expression was not affected by OA in rat vascular smooth muscle cells [33] and was downregulated by oleate in macrophages RAW264.7 cells [34] and small intestine enterocytes [35]. These discrepancies may be due to the different cell types and experiment designs. Second, we found that CD36 knockdown with CD36 siRNA reversed the OA-induced promotion of HC11 proliferation, increase in EdU positive cells percentage, and alteration in the expression of proliferative markers (CyclinD1/3, PCNA and p21). Together, these observations suggested that CD36 was involved in OA-stimulated HC11 proliferation and pubertal mammary gland development. Similarly, it has been reported that CD36 is involved in OA detection by murine olfactory system [36] and in OA-induced smooth muscle foam cell formation [33]. It should be noted that the GPR40 and GPR120 receptors also both recognize LCFAs [37, 38]. In our preliminary experiment, we examined the effects of OA on the expression of GPR120 and found that OA had no effect on GPR120 expression (data not shown). While the possible role of GPR40 in OA-stimulated HC11 proliferation need to be further investigated in the subsequent study.

It has been demonstrated that CD36 activation by OA can lead to increase of [Ca^2+^]_i_ level in olfactory neurons [36], taste bud cells [39, 40] and immune-competent cells [41]. Meanwhile, there is much evidence indicating that Ca^2+^ signals are involved in the control of cell proliferation [42, 43]. Accordingly, in the present study, we found that OA resulted in the remarkable increase in the [Ca^2+^]^i^ concentration. However, the OA-induced [Ca^2+^]^i^ elevation was eliminated by CD36 knockdown, suggesting the link between CD36 and the [Ca^2+^]^i^ signal in this process. In addition, the chelation of [Ca^2+^]^i^ reversed the promotion of HC11 proliferation and change in proliferative marker expression induced by OA. These findings demonstrated that the activation of the CD36-[Ca^2+^]^i^ signaling pathway might participate in OA-induced pro-proliferative effects on HC11 cells.

Numerous studies have demonstrated that the activation of the PI3K/Akt signaling pathway is involved in regulating mammary epithelia proliferation and mammary gland development [9, 44–46]. In agreement with previous results, we found that the PI3K/Akt signaling pathway was activated by OA treatment in both HC11 cells and the mammary gland of mice. Interestingly, the activation of the PI3K/Akt signaling pathway was abolished by CD36 knockdown and the chelation of [Ca^2+^]_i_, suggesting that the PI3K/Akt signaling pathway was the downstream target of the CD36-[Ca^2+^]_i_ signaling pathways. Moreover, the inhibition of the PI3K/Akt signaling pathway with WT eliminated the OA-induced stimulation of HC11 proliferation and alteration in proliferative markers expression. Together, these results provided evidence that OA stimulated HC11 proliferation and mammary duct growth via the activation of CD36-[Ca^2+^]_i_ and the linked intracellular PI3K/Akt signaling pathway.

In conclusion, our findings showed that OA stimulated HC11 cells proliferation and mammary gland development in peripubertal mice, which was associated with the activation of CD36-[Ca^2+^]_i_ and PI3K/Akt signaling pathway. These results provide new insights into the nutritional regulation of peripubertal mammary gland development by dietary monounsaturated LCFA (OA) and suggest the potential application of OA as a functional food in promoting peripubertal mammary gland development.

## MATERIALS AND METHODS

### Reagents and antibodies

Oleic acid (OA, #O1008, purity ≥ 99%) used in the *in vitro* study was purchased from Sigma-Aldrich (St. Louis, MO, USA). OA used in the *in vivo* study was purchased from Chengdu Chemical Technology Co., Ltd (Chengdu, Sichuan, China, purity ≥ 95%). PI3K/Akt inhibitor Wortmannin (WT) and MTT assay kit was purchased from Beyotime Biotechnology Inc (Shanghai, China). RPMI-1640 medium and fetal bovine serum (FBS) were purchased from Gibco BRL (Gaithersburg, MD, USA). DAPI was purchased from Biosharp (Hefei, Anhui, China). 5-ethynyl-2’-deoxyuridine (EdU) incorporation assay kit was purchased from Ribobio Biological Technology Co., Ltd (Guangzhou, Guangdong, China). CD36 siRNA was purchased from Suzhou Zimmer Genomics Technology Co., Ltd (Suzhou, Jiangshu, China). Lipofectamine 2000 was purchased from Life Technologies (Carlsbad, CA, USA). Antibodies against Cyclin D1 (#2922), p21 (#2947), phospho-Akt _Ser473_ (p-Akt _Ser473_, #4060) and Akt (#9272) were purchased from Cell Signaling Technology Inc (Danvers, MA, USA). Antibody against CD36 (#ab133625) was bought from Abcam (Cambridge Science Park, UK). Antibodies against β-actin (#bs-0061R) and PI3K (#bs-0128R) were purchased from Beijing Bioss Biotechnology Co., Ltd (Beijing, China). Antibody against phospho-PI3K_Tyr508_ (p-PI3K_Tyr508_, # sc-12929) was purchased from Santa Cruz Biotechnology Inc (Santa Cruz, CA, USA). The goat-anti-rabbit FITC conjugated secondary antibody (bs-0295G-FITC) were purchased from Beijing Bioss Biotechnology Co., Ltd (Beijing, China). The goat anti-rabbit HRP conjugated secondary antibody (#BS13278) and rabbit anti-goat HRP conjugated secondary antibody (#BS30503) were purchased from Bioworld Technology, Inc. (St. Louis Park, MN, USA).

### Cell Culture and Treatment

Mouse mammary epithelial cell line HC11 cells (Action-award Biological Technology Co., Ltd, Guangzhou, Guangdong, China) were seeded at a density of 25,000 cell/ cm^2^ and cultured in RPMI-1640 medium supplemented with 10% FBS, 100 U/mL penicillin and 100 μg/mL streptomycin in 96-well plated for proliferation assay, while in 6-well plate for ICC, Western blot and real-time PCR experiments. The medium were changed every two days and the cells reached about 100% confluence at day 4. All treatment groups were performed on the same plate for each replicate, with at least three replicates for each treatment group. HC11 was incubated with various concentrations (0, 12.5, 25, 50, 100, and 200 μM) of OA for 4 days to investigate the dose effect of OA. Meanwhile, HC11 cells transfected with CD36 siRNA were incubated with or without 100 μM OA for 4 days to elucidate the role of CD36 in OA-mediated HC11 proliferation. In addition, the cells were cultured for 2 days before the [Ca^2+^]_i_ assay. The cells were incubated with 100 μM OA and/or 2 μM chelator BAPTA-AM (Sigma-Aldrich) for 4 days to determine the role of [Ca^2+^]_i_ in OA-mediated HC11 proliferation. Furthermore, the cells were cultured with 100 μM OA and/or PI3K inhibitor Wortmannin for 4 days to explore the role of PI3K/Akt signaling pathway in OA-mediated HC11 proliferation.

### MTT Assay and EdU Incorporation Assay

The proliferation of HC11 were assessed at day 4 by using MTT Cell Proliferation and Cytotoxicity Assay Kit and Cell-Light ^TM^ EdU imaging detecting kit as previously described [9]. Briefly, for MTT assay, the HC11 cells in 96-well plate were incubated at 37°C with 10 μL MTT (5 mg/ml) in each well for 4 h, and then incubated at 37°C with 100 μL Formazan lysate for 4 h. The number of viable cells were assessed by measuring the absorbance at 570 nm using a Synergy 2 Multi-Mode Reader (Bio Tek Instruments, Inc., Winooski, VT, USA). For EdU incorporation assay, HC11 in 96-well plate was incubated with 50 μM EdU for 2 h. After washed with PBS for 3 times, the cells were fixed with 4% paraformaldehyde for 30 min at room temperature, and then stained with Apollo ^®^ staining reaction solution and Hoechst 33342 reaction mixture in dark at room temperature for 30 min. The cells were captured with a Nikon Eclipse Ti-s microscope (Nikon Instruments, Tokyo, Japan). The percentage of EdU-positive nuclei to total nuclei was analyzed in five high-power fields per well.

### Transfection of HC11 with siRNA

HC11 cells were transfected with 4 pmol siRNA specific for CD36 or scrambled siRNA using Lipofectamine 2000 for 6 h according to the manufacturer’s instructions. Subsequently, the cells were treated with or without OA for 2 d. Then, the cells were transfected (6 h) and incubated with or without OA (2 d) once again. The knockdown efficiency of CD36 were confirmed by Western blot.

### Immunocytochemistry

The immunocytochemical staining of Cyclin D1 and p21 in HC11 was performed as previously described [9]. Briefly, HC11 in 6-well plate was fixed with 4% paraformaldehyde, permeabilized with 0.4% Triton X-100, and then blocked with PBS containing 1% goat serum for 1 h at room temperature. The cells were then incubated with CyclinD1 (1:800) or p21 (1:800) or PBST (negative control) at 4°C overnight. Thereafter, membranes were incubated with the goat-anti-rabbit FITC-conjugated secondary antibodies (1:1000) for 1 h at room temperature. The cells were observed and captured with a Nikon Eclipse Ti-s microscope, and the fluorescence intensity were quantified with Nis-Elements BR software (Nikon Instruments, Tokyo, Japan).

### Measurement of [Ca^2+^]^i^

[Ca^2+^]_i_ was measured by calcium fluorometry using fluo-8 AM as previously described [47]. Briefly, the HC11 transfected with CD36 siRNA or scrambled siRNA was seeded in a 24-well plate and cultured for 2 days, when they reached more than 50% confluence. The cells were washed twice with Hank’s balanced salt solution (HBSS, pH 7.2-7.4) and incubated with 10 μM fluo-8 AM at 37°C. After 1 h of incubation, the cells were washed twice with HBSS, and the calcium response assay was initiated by manual addition of 100 μM OA equipped with Nikon Eclipse Ti-s microscopy. Fluorometric data were acquired at excitation and emission wavelengths of 490 and intensity at 525 nm (490/525 nm) for every 5 s interval over a 180 s period.

### Western blot analysis

The protein of HC11 cells and mammary glands was extracted and western blot analysis was conducted as previously described [48]. Equivalent amounts of protein (25 μg) were separated by 10% SDS-PAGE and the samples were transferred onto nitrocellulose membranes (BioRad, Hercules, CA, United State). Primary antibodies used included CyclinD1 (1:2000), p21 (1:2000), CD36 (1:2000), Akt (1:2000), p-Akt _Ser473_ (1:2000), PI3K (1:2000), p-PI3K_Tyr508_ (1:800) and β-actin (1:1000). The second antibodies used were goat anti-rabbit HRP conjugated antibody (1:50000) and rabbit anti-goat HRP conjugated antibody (1:50000). Densitometry analysis was perform using image J software and band density was normalized to the b-actin.

### Real-time quantitative PCR

After the HC11 cells were cultured with various treatments for 4 days, the mRNA expression of Cyclin D1, Cyclin D3 and PCNA were examined by real-time quantitative PCR as previously described [9]. Briefly, total RNA was extracted from the HC11 cells by using an RNA extraction kit (Guangzhou Magen Biotechnology Co., Ltd, Guangdong, China) and cDNA was synthesized from 2 μg of total RNA by the M-MLV Reverse Transcriptase (Promega, Madison, WI, USA) and random primers oligo-dT18. β-actin was used as a candidate housekeeping gene. Real-time quantitative PCR was carried out in Mx3005p instrument (Stratagene, La Jolla, CA, USA) by using SYBR Green Real-time PCR Master Mix reagents (Toyobo Co., Ltd., Osaka, Japan) and both sense and antisense primers (200 nM for each gene). Primer sequences (with their respective PCR fragment lengths) were shown in Table 1.

**Table 1:** Primer sequences used for real-time quantitative PCR

Gene	Forward (5′–3′)	Reverse (5′–3′)	Amplification size (bp)
β-actin	GGTCATCACTATTGGCAACGAG	GAGGTCTTTACGGATGTCAACG	142
Cyclin D1	CTGAAGGCTCGCGGAATAAAA	GCAAGTTGTGGGCAGCAATA	145
Cyclin D3	CGAGCCTCCTACTTCCAGTG	GGACAGGTAGCGATCCAGGT	150
PCNA	TTTGAGGCACGCCTGATCC	GGAGACGTGAGACGAGTCCAT	135

### Animal and *in vivo* study

All animal experiments were conducted with the permission number of SYXK (Guangdong) 2014-0136, and animal care procedures were performed in accordance with the guidelines for the care and use of animals approved by The Animal Ethics Committee of South China Agricultural University.

Twenty C57BL/6 female mice (3-week-old) purchased from Guangdong Medical Laboratory Animal Center were acclimated for one week and then randomly divided into 2 groups: a control group, which was fed a control diet (formulated according to the commercial mice diet with the feedstuff purchased from Guangdong Medical Laboratory Animal Center); a OA group, which was fed a control diet containing 2% (w/w) OA. The calorie content in the diet of control and OA group is balanced with the same total energy. The mice were housed in environmentally controlled rooms on a 12-h light-dark cycle with free access to food and water. The body weight and food intake were recorded weekly. At the end of 5 weeks of treatments, mice were anaesthetized by carbon dioxide. The blood was collected and incubated at 37°C for 1 h and then centrifuged at 1,500 g for 20 min. Then the serum was collected and stored at –20°C for further determination of the serum levels of IGF-1 and estradiol (E2). The fourth and part of the fifth pair of mammary gland was rapidly isolated and weighed. The left side of the mammary gland was fixed for whole mount staining. The right side of the mammary gland was frozen in liquid nitrogen and stored at –80°C until further Western blot analyses.

### Whole mount staining

The whole mount staining of the mammary gland was performed as previously described [9].

### Statistical analysis

All data are presented as means ± standard error of the mean (S.E.M.). For cell culture studies, three independent experiments were conducted with at least 3 parallel measurements in each experiment. In the mouse feeding trial, individual animal was considered as an experimental unit and ten mice in each group were used. Statistical analysis was performed using SigmaPlot 12.5 (Systat Software, Inc., San Jose, CA, USA). Differences between means were determined using two-tailed Student’s *t*-test or One Way ANOVA with a Dunnett’s post-hoc test and a confidence level of *P* < 0.05 was statistically significant.

## Abbreviations

Akt: protein kinase B
[Ca2^+^]^i^: intracellular calcium
CD36: fatty acid translocase
E2: estradiol
EdU: 5-Ethynyl-2’-deoxyuridine
HC11: HC11 mouse mammary epithelial cells
IGF-1: insulin-like growth factor-1
OA: oleic acid
LCFA: long-chain fatty acids
MCFA: medium-chain fatty acids
MUFA: monounsaturated fatty acid
p21: cyclin-dependent protein kinase inhibitor 21
PUFA: polyunsaturated fatty acids
PCNA: proliferating cell nuclear antigen
PI3K: phosphatidylinositol 3-kinase
siRNA: small interfering RNA
TDE: terminal duct end
WT: wortmannin
